# Travel Is a Key Risk Factor for Malaria Transmission in Pre-Elimination Settings in Sub-Saharan Africa: A Review of the Literature and Meta-Analysis

**DOI:** 10.4269/ajtmh.18-0456

**Published:** 2020-08-17

**Authors:** Sundus Ahmed, Richard Reithinger, Stephen K. Kaptoge, Jeremiah M. Ngondi

**Affiliations:** 1 Institute of Public Health, University of Cambridge, Cambridge, United Kingdom;; 2 RTI International, Washington, District of Columbia;; 3 RTI International, Dar es Salaam, Tanzania

## Abstract

By sustaining transmission or causing malaria outbreaks, imported malaria undermines malaria elimination efforts. Few studies have examined the impact of travel on malaria epidemiology. We conducted a literature review and meta-analysis of studies investigating travel as a risk factor for malaria infection in sub-Saharan Africa using PubMed. We identified 22 studies and calculated a random-effects meta-analysis pooled odds ratio (OR) of 3.77 (95% CI: 2.49–5.70), indicating that travel is a significant risk factor for malaria infection. Odds ratios were particularly high in urban locations when travel was to rural areas, to more endemic/high transmission areas, and in young children. Although there was substantial heterogeneity in the magnitude of association across the studies, the pooled estimate and directional consistency support travel as an important risk factor for malaria infection.

## INTRODUCTION

It is widely recognized that human mobility influences the spread of infectious diseases,^1^ and evidence suggests that human movement was partly responsible for the failure in the previous global malaria eradication campaign.^2^ Over the last century, following widespread malaria control/elimination strategies, transmission risk varies markedly between and within countries; thus, travel across different transmission settings markedly influences malaria vulnerability, especially in areas with high malaria receptivity.^3^ In the past, malaria and travel largely focused on international travelers to countries or areas at risk of transmission arriving from countries of no risk, who were more susceptible to severe malaria because they lacked immunity.^4^ Tremendous progress in the reduction in malaria morbidity and mortality has been achieved in the last decade, with 21 countries projected to attain elimination by the year 2020.^5,6^ Therefore, imported malaria has become an important risk factor in formerly malaria-endemic countries that had attained elimination and in low transmission countries that are targeted for elimination. These settings continue to experience thousands of malaria cases every year through importation, resulting in increased morbidity and mortality, a substantial burden to the health system, and occasionally secondary transmission.^7^ Findings from analyses of international population movements using census-based migration and reported malaria data suggested that certain groups of countries were much more strongly connected by relatively high levels of population and infection movement than others.^2,7^ Therefore, in malaria post-elimination and pre-elimination settings, imported malaria remains an important threat to the gains that have been achieved.^8,9^

Notwithstanding, the literature on travel in the context of malaria transmission is limited, and few studies have investigated this relationship, particularly in sub-Saharan Africa. To examine the role of travel as a potential risk factor for malaria infection and imported malaria at large, we conducted a literature review and meta-analysis of studies investigating the association between history of travel and malaria infection status in sub-Saharan Africa.

## METHODS

### Literature review.

The selection of studies was conducted using the Preferred Reporting Items for Systematic Reviews and Meta-Analyses guidelines. A literature search was conducted using PubMed in September 2019 to identify studies that investigated the relationship between travel and malaria using combinations of the Medical Subject Heading (MeSH) terms “travel,” “risk factor,” and “malaria.” Articles were screened first by title, then by abstract, and then by full text for inclusion in the review. References of key articles were also reviewed to identify additional relevant studies. Inclusion criteria included 1) assessment of the relationship between travel and malaria infection in an endemic setting using odds ratio (OR) as the measure of association; 2) history of travel, defined as an exposure with any geographic or temporal variation used (i.e., travel outside the village/country to an area of higher transmission and travel in the past 1 month/2 months); and 3) study location in sub-Saharan Africa. Exclusion criteria included 1) no assessment of a relationship with travel (i.e., studies looking at clinical management, treatment, interventions, giving travel advice, and using a measure of association other than OR); 2) history of travel not defined as a specific exposure or risk factor (i.e., studies looking only at travel/migration patterns and importation burdens); and 3) study location in endemic country outside of sub-Saharan Africa. Two authors (S. A. and S. K. K.) independently screened the titles and abstracts of the articles using the inclusion/exclusion criteria. Any discrepancies were discussed with the third author (J. M. N.), and decision on inclusion was made jointly.

### Statistical analysis.

Measures of association (ORs with 95% CIs) and key study or participant characteristics (including geographic location, study design, sample population, sampling/recruitment methods, sample size, malaria case definition, definition of travel history, and confounders adjusted for) were extracted for the meta-analysis. For a few studies that only reported separate OR estimates for predefined subgroups (e.g., by gender), a single estimate was first calculated for the study by inverse-variance weighted fixed-effect meta-analysis and further used in the overall meta-analysis. A random-effects meta-analysis was conducted using the most adjusted OR from each study using Stata version 13 (Stata Corporation, College Station, TX). Between-study heterogeneity was assessed using the *I*^2^ statistic,^10^ and association with study-level characteristics was assessed using meta-regression. Sensitivity analysis was undertaken by stratifying the meta-analysis by study characteristics, including geographical region type of malaria tests, and level of adjustment of confounders.

## RESULTS

### Studies included in meta-analysis.

**Figure 1.**
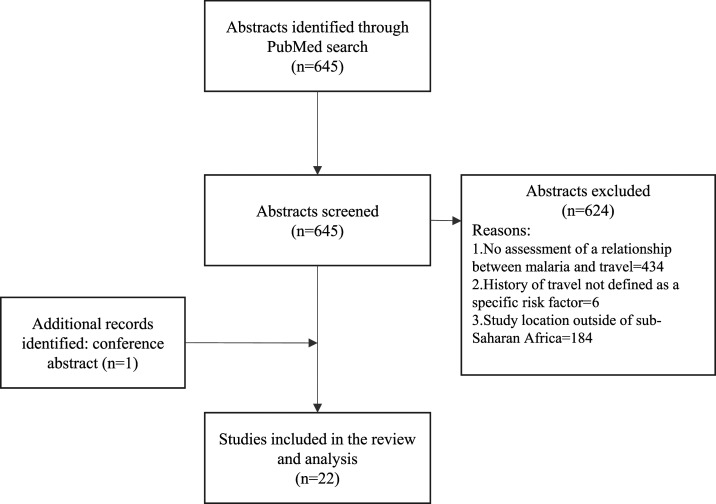
Flowchart of the studies included in the review and reasons for exclusion.

The literature search identified 645 publications. Following title, abstract, and full-text review, only 22 studies from the PubMed search and one study published as a conference abstract met the inclusion and exclusion criteria, and thus were retained for the meta-analysis (Figure 1, Table 1).

**Table 1:** Summary of studies assessing travel as a risk factor for malaria infection

Location (date)	Study design (*N*)	Sample population	Sampling/recruitment method	Case definition (malaria diagnosis)	Exposure (definition of travel history)	OR (95% CI), *P*-value	Confounders adjusted for		Reference
BotswanaTubo village (June 2012)	Retrospective case–control (*N* = 71)	Inhabitants of one village	Interviewed 71 household heads	Self-reported	Travel outside village in the past 8 months	2.70 (1.00–7.26)	None		11
Burkina FasoOuagadougou (November–December 2002)	Case–control (*N* = 873)	Patients presenting to health facilities in Ouagadougou (one health facility was selected from each of three areas)	200 patients with fever + 200 non-fever controls selected from each health facility	*Plasmodium* infection confirmed by blood smears	Travel to a rural area in the past 90 days	1.14 (0.70–1.90), 0.6	Participants matched for age and residence		12
Burkina FasoOuagadougou (urban areas) (April–May and September–October 2004)	Cross-sectional (*N* = 3,354)	Children aged 6 months to 12 years in select households across eight areas/four ecological strata	Residents of Ouagadougou for ≥ 5 years were included; two surveys conducted in separate time periods to account for seasonal variations	*Plasmodium* infection confirmed by blood smears	Travel outside of Ouagadougou in the past month	1.11 (0.78–1.58), 0.593	None		13
Ethiopia Oromia region (June–October 2011)	Unmatched case–control (*N* = 560)	Adult patients (18+ years old) presenting to Bulbula health facility	Adult patients presenting with fever or a history of fever in the past 72 hours and tested for malaria	*Plasmodium* infection confirmed by blood smears or multispecies RDT	Travel overnight away from home village in the past 30 days	AOR = 1.64 (1.07–2.52), = 0.02	Gender, age, SES, household characteristics, and use of ITNs		14
Ethiopia Dabat district (high-altitude villages) (August 2012–May 2013)	Unmatched case–control (*N* = 1,455)	Patients presenting to four health facilities	Patients ≥ 15+ years presenting to the health facilities, permanent residents of the Dabat district, and had a history of fever ≤ 72 hours tested for malaria	*Plasmodium* infection confirmed by blood smear or multispecies RDT	Travel away from home in past month	AOR = 2.01 (1.56–2.58), < 0.0001	Sex, occupation, plants used for fencing, forests near house, borehole near house, and ITNs availability		15
Ethiopia Hadiya zone (May–June 2014)	Cross-sectional (*N* = 411)	Patients presenting to 12 health facilities in Hadiya Zone (low transmission area)	Facilities randomly selected and participants selected by systematic sampling of febrile patients	*Plasmodium* infection confirmed by blood smear	Travel to a malaria-endemic area in the past 30 days	AOR = 2.59 (1.24–5.38)	Gender, ever heard about malaria, had home visit by health extension worker, knowledge score level, bed net ownership, practice score level, family size, and distance from stagnant water		16
Ethiopia Amhara region (August–September 2014)	Case–control (*N* = 30,712)	Household members aged > 6 months	Households in six villages across eight districts of region selected because of having higher transmission surveyed	Positive RDT	Spent at least one night away from home in the past month	< 10 years old: AOR = 10.16 (1.18–87.58). > 10 years old: AOR = 6.07 (2.48–14.81)	Gender, age, occupation of the household head, village, and malaria risk factors (vector control methods, febrile, taking antimalarial drugs in the last 2 weeks, > 1 RDT-positive individual in the household, and ≥ 1 individual in the household spent ≥ 1 night away from home in the last month)		17
Ethiopia Lake Tana surroundings (northwest, ∼1,750–2,500 m above sea level) (October 2016–June 2017)	Matched case–control (*N* = 606)	Patients aged ≥ 5 months from 11 villages of Gondar Zuriya district and one village of Dembia district	Patients resident in the villages were invited to participate + controls (matched for age and gender)	Symptoms of malaria or fever in previous 48 hours + *Plasmodium* infection confirmed by blood smear	Travel to malarious lowlands in the past month	AOR = 7.32 (2.40–22.34), < 0.0001	Matched for age and gender; adjusted for marital status, occupation, education status, household wealth index, roofing materials, flooring materials, travel history within study area, household member travel to malarious lowlands, received health information on malaria, LLIN ownership, household elevation, and house proximity to health center/water body/Lake Tana		18
Ethiopia (August–December 2014)	Matched case–control (*N* = 520)	Residents of seven villages > 2,000 m above sea level of Tahtay-Maychew district	Cases were randomly selected across villages + controls (three per case) were randomly selected from nearby households	Positive RDT	Travel outside of home village to a malaria-endemic area in the past month	AOR = 11.40 (6.91–18.82), < 0.001	Matched by gender and village; adjusted for age, education level, owns livestock, and main occupation (agricultural vs. nonagricultural)		19
Ghana Accra and Kumasi (October 2002–February 2003)	Cross-sectional (*N* = 3,525)	Children aged 6 months to 5 years	Children selected from households in 23 communities selected for being close to/far away from urban agricultural sites	*Plasmodium* infection confirmed by blood smear	Travel to a rural area in the past 3 weeks	Accra: NS Kumasi: 7.0, 0.0005	SES, being anemic, education level of caregiver, use of windows/door nets, use of mosquito coils, and the presence of ceiling in house		20
Ghana Kumasi (two neighboring communities, Moshie Zongo and Manhyia) (April–May 2005)	Cross-sectional (*N* = 296)	Children aged < 5 years in select households	Systematic selection of households until 10 children reached per cluster; all children present in household were invited to participate	Positive RDT	Travel to a rural area in the past 3 weeks	AOR = 23.45 (3.28, 167.75)	Education level of caregiver > middle school, ethnic group, SES, use of repellent/coils/spray, distance to nearest health facility, and age-group		21
Ivory Coast Abidjan (Yopougon municipality) (September 2002)	Cross-sectional (*N* = 812)	Patients at multiple health facilities (one selected per geographical zone of the municipality—urban and peri-urban areas)	200 patients currently with fever + 200 non-fever controls selected from each health facility	*Plasmodium* infection confirmed by blood smears	Travel to a rural area in the past 90 days	1.75 (1.25–2.45), < 0.001	Matched for age and residence		22
Kenya (Western highlands) (1998–2002)	Case–control (*N* = 12,999)	Kericho tea estate workers	Interview of asymptomatic persons on Kerenga tea estate from 1999 to 2000 and outpatients presenting to Kerenga health center with symptoms suggestive of malaria from 1998 to 2002	*Plasmodium* infection confirmed by blood smears	Travel away from the Kericho tea estates in past 2 months	Well persons: 1.59 (1.20–2.1). Outpatients: 2.38 (2.17–2.6)		None	23
Malawi Blantyre (low transmission, urban area) (2010)	Matched case–control (*N* = 767)	Children aged < 5 years presenting to a health facility in Ndirandi (township in Central Blantyre)	Febrile children and children positive for malaria at health facility + controls children presenting to clinic and randomly selected when visiting households	Axillary temperature of 37.5°C or a history of fever within the last 48 hours and *Plasmodium* infection confirmed by blood smears	Travel to rural areas	6.66 (4.79–9.61)		Matched for age ± 6 months and gender; adjusted for SES, household proximity to garden, standing water and proximity to river, marital status, and woman employment status	24
Malawi Blantyre (four urban and two peri-urban areas) (April 2012–October 2015)	Matched case–control (*N* = 473)	Children aged 6 months to 5 years presenting to 6 health facilities (four urban and two peri-urban)	Febrile children testing positive for malaria infection (blood smear and PCR) + controls (two controls per case)	Temperature ≥ 37.5°C or a history of fever within the last 48 hours, malaria-confirmed blood smears for all *Plasmodium* species and PCR for *Plasmodium falciparum*	Travel for at least one night outside city limits in the past month	Overall: 2.35 (1.04–5.3), 0.04. Urban: AOR = 2.36 (1.31–4.2). Peri-urban: AOR = 1.17 (0.64–2.15), < 0.02		Matched by age category (6–24 or 25–59 months), zone of residence within the health facility catchment area, and time of diagnosis (within 4 weeks); and adjusted for having electricity in household and tertiary education	25
Mozambique Maputo city (urban, peri-urban, and rural areas)	Case–control (*N* = 645)	Patients presenting to 28 public outpatient health facilities (urban, peri-urban, and rural)	Patients with fever presenting for the first time and weighing more than 5 kg	Temperature ≥ 37.5°C or a history of fever in the past 24 hours, and *Plasmodium* infection confirmed by blood smears	Travel for at least one night outside city limits in the past 3 months	Overall: AOR = 1.82 (1.08–3.07), 0.025. Urban: 3.93 (1.56–9.89)		Age-group (≥ 5 or < 5 years old), fever at enrollment, house close to water, health facility location (peri-urban vs. urban), and health facility location (rural vs. urban)	26
Namibia North central area along border with Angola (December 2012–July 2014)	Case–control (*N* = 786)	Patients presenting to 46 health facilities across Engela, Outapi, and Oshikuku health districts	Patients with confirmed malaria resident in study area + controls (RDT-negative individuals residing in randomly selected households)	Positive RDT	Travel in the past 4 weeks	AOR for travel to Angola within gender strata: male = 43.58 (2.12–896). Female = 1.65 (0.36–7.47)		Age-group, location slept the previous night, predicted travel time to clinic, distance from the Angolan border, enhanced vegetation index, total rainfall in prior month, and district	27
UgandaKabale (highland site ∼2,200 m above sea level) and Rukungiri (highland fringe site ∼1,500 m above sea level) (2007)	Matched case–control (*N* = 104 highland, *N* = 168 highland fringe)	Patients presenting to two health facilities	Patients presenting malaria symptoms and residents in study area + controls (one per case)	Positive RDT	Travel for at least one night in the past 4 weeks outside subcounty of residence (classified as travel to area of low/equal transmission risk or high transmission risk)	Highland: AOR = 4.7 (1.4–16.3), 0.01 (overall); AOR = 1.5 (0.3–7.9), 0.7 (low/equal risk); AOR = 6.9 (1.4–33.1), 0.01 (high risk). Highland fringe: AOR = 2.1 (1.1–3.9), 0.04 (overall); AOR = 2.9 (1.6–5.2), < 0.0001 (low/equal risk); AOR = 0.7 (0.1–3.7), 0.7 (high risk)		Matched for age, gender, village of residence, and date of presentation; adjusted for SES and altitude	28
Swaziland (January 2010–June 2014)	Cross-sectional (*N* = 11,376)	Patients presenting to multiple health facilities across country	Confirmed malaria cases at health facility were followed up at the household level within 7 days, and “fever screening” was conducted to identify additional nearby cases	Positive RDT and/or microscopy	Travel within or outside country in past 8 weeks	Overall: AOR = 63.4 (16.2–311.07), < 0.0001. Travel within country: AOR = 3.56 (2.72–4.61), < 0.0001. Travel outside country: AOR = 31.76 (25.76–39.26), < 0.0001		Age, occupation, gender, and nationality	29
Tanzania Dar es Salaam (June–August 2003)	Cross-sectional (*N* = 1,498)	Patients presenting to select health facilities/schools (1–2 in each of four zones)	200 patients with fever + 200 non-fever controls selected from each health facility	Fever ≥ 37.5°C or a history of fever in the past 36 hours, *Plasmodium* infection confirmed by blood smears	Travel to rural area in past 3 months	Age ≤ 5 years: AOR = 3.62 (1.48–8.88), < 0.05. > 5 years old: AOR = 2.80 (1.23–6.37), < 0.01		Matched for age and residence	30
TanzaniaZanzibar (2015)	Case–control (*N* = 18,640)	Patients presenting to 157 public and 77 private health facilities in Zanzibar	Patients with confirmed malaria were followed up at the household level	Positive RDT	Travel for at least one night outside Zanzibar in the past 30 days	9.8 (8.3–11.6)		Age-group, gender, slept under LLINs the previous night, history of fever in the last 2 weeks, household characteristics, and net density	31
TanzaniaZanzibar (May–June between 2003 and 2015)	Case–control (*N* = 3,734)	North A district in Unguja Island and Micheweni district in Pemba Island	Shehias and households were randomly selected; multiple surveys were conducted at the household level	Positive blood smear, RDT, PCR, or serological testing	Travel within or outside Zanzibar in the past 30 days	Travel within Zanzibar: AOR = 1.1 (0.7–1.6). Travel outside Zanzibar: AOR = 70.2 (50.0–100.6)		Gender, sleeping under bed net, and using indoor residual spraying	32

AOR = adjusted odds ratio; ITNs = insecticide-treated nets; LLINs = long-lasting insecticidal nets; OR = odds ratio; RDT = rapid diagnostic test; SES = social economic status.

A summary of the study locations, design, sample selection, definition of travel, reported ORs, and confounding factors adjusted is shown in Table 1. The 22 studies included in this review were carried out in Botswana, Burkina Faso (2), Ethiopia (6), Ghana (2), Ivory Coast, Kenya, Malawi (2), Mozambique, Namibia, Uganda, Swaziland, and Tanzania (3). They primarily reported results from study sites with low transmission intensity, and all used logistic regression modeling to test the association of history of travel (and other covariates) with malaria infection status. All studies used either a case–control or cross-sectional design, but some differed in their case ascertainment methods and geographical/temporal criteria for defining the travel variable. Most of the studies recruited patients diagnosed with malaria at particular health facilities for inclusion; seven studies surveyed randomly selected households across the study area; one study in Botswana^11^ looked at all inhabitants of the study site, and one study in Ethiopia^19^ looked at all current residents of the seven villages selected. Two studies used data collected from active surveillance systems (Tanzania^31^ and Swaziland^29^). Most studies defined history of travel as having travelled overnight outside of the home village or area in the past month. A handful of studies restricted participants to a particular age criteria: both of the Malawi and Ghana studies looked only at children aged < 5 years^20,21,24,25^; one of the Burkina Faso studies looked only at children aged < 12 years,^13^ and one of the Ethiopia studies looked only at adults aged 18 years and older.^14^ Some of the studies further specified if travel was to areas that were rural,^12,20–22,24,30^ malaria endemic,^16,18,19^ or had an equal/lower or higher transmission intensity,^28^ but most of the studies did not.

### Meta-analysis results.

**Figure 2.**
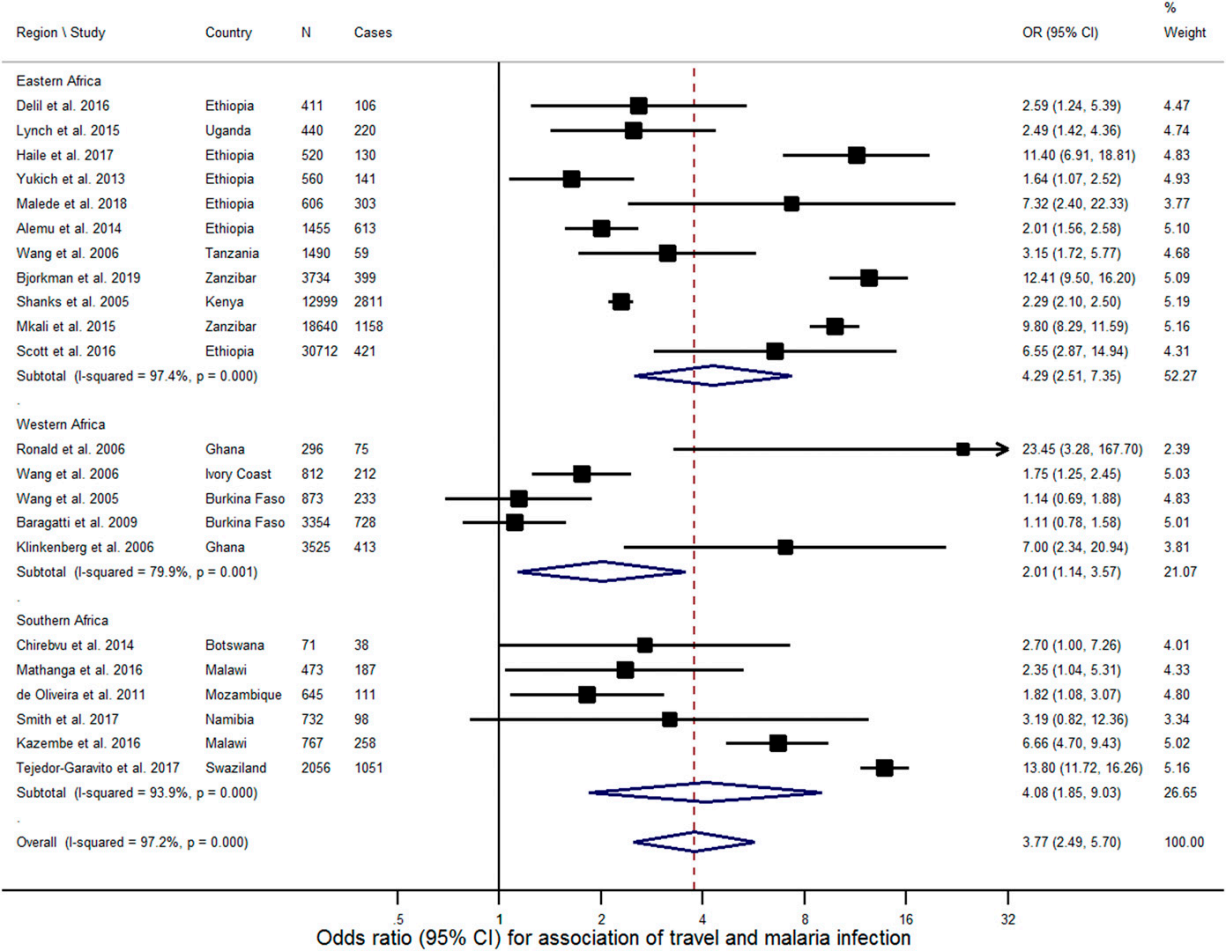
Forest plot and measures of association for meta-analysis. This figure appears in color at www.ajtmh.org.

**Figure 3.**
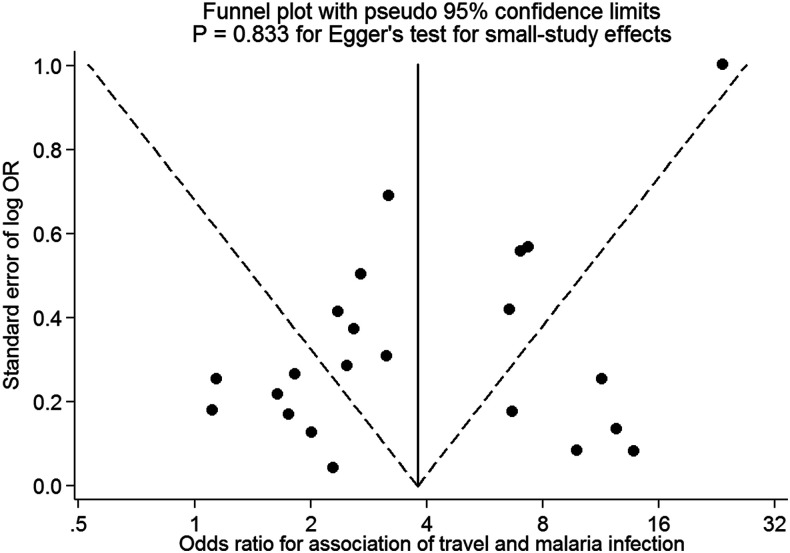
Funnel plot assessing potential for small-study effects.

A forest plot of the meta-analysis, with studies grouped by geographical location, is presented in Figure 2. Overall, all but three of the studies^12,13,27^ found that history of travel was significantly associated with malaria infection. The pooled OR was 3.77 (95% CI: 2.49–5.70)—indicating the odds of having malaria infection were almost 4-fold higher among individuals with a travel history than to those with none. There was substantial heterogeneity across studies (*I*^2^ = 97% [95% CI: 97%, 98%]) that was only partly explained by study-level characteristics such as geographic location, method of malaria testing, extent of adjustment for confounders, and duration of travel (meta-regression residual *I*^2^ = 70% [44%, 84%]). There was no evidence of small study effects, including potential publication bias, as visually judged from symmetry of funnel plot (Figure 3) and confirmed with formal statistical test (*P* = 0.833 for Egger’s test of small-study effects).

The ORs from the individual studies ranged from 0.7 in Rukunguri, Uganda (for people of the highland fringe site travelling to areas of higher transmission intensity),^28^ to 70.2 in Zanzibar, Tanzania (for those travelling outside of the island).^32^ Travel away from the Namibia study site to Angola in the past 4 weeks had a particularly high OR for males (adjusted odds ratio [AOR] = 43.58, 95% CI: 2.12–896) as opposed to females (AOR = 1.65, 95% CI: 0.36–7.47).^27^ Similarly, travel within or outside Swaziland in the past 8 weeks had a particularly high OR overall (AOR = 63.4, 95% CI: 16.2–311.07, *P* < 0.0001), most of which was accounted for by travel outside the country (AOR = 31.76, 95% CI: 25.76–39.26, *P* < 0.0001) as opposed to travel within the country (AOR = 3.56, 95% CI: 2.72–4.61, *P* < 0.0001).^29^ Travel to an area of higher malaria risk from the highland site in Uganda also had a greater OR (AOR = 6.9, 95% CI: 1.4–33.1, *P*-value = 0.02) as opposed to an area of lower or equal risk (AOR = 1.5, 95% CI: 0.3–7.9, *P*-value = 0.7).^28^ Studies in Mozambique and Malawi found travel history outside city limits and malaria infection to have a strong association, specifically for urban populations (OR = 3.93, 95% CI: 1.56–9.89 and AOR = 2.36, 95% CI: 1.31–4.2, respectively).^25,26^ The two Burkina Faso studies calculated slightly increased odds for those travelling contracting malaria, but these odds were not statistically significant.^12,13^ In Ghana, a significant association between travel and malaria was only observed for people in the city of Kumasi but not in Accra.^20^

## DISCUSSION

The results of the meta-analysis strongly suggest that travel is a key risk factor for acquiring malaria infection, particularly in urban areas and when travel is to rural/malaria-endemic areas or areas of higher transmission intensity than the origin location. The Swaziland and Zanzibar surveillance studies hold the second greatest weight in our meta-analysis (based on the number of participants and cases) and found people with a history of travel to be almost 14 and 10 times more likely to have malaria infection than those with no history of travel, respectively.^29,31^ As described earlier, only three studies in the current analysis specified that the destination of travel was to an endemic area and only one looked at the transmission intensity at the destination. Six studies in the current analysis looked specifically at travel to rural areas as the cause of malaria importation into urban and peri-urban areas, of which all but one found a significant association.^20–22,24,30^ This is in agreement with a previous analysis that suggested that travel to rural areas is a major source of malaria transmission in urban areas.^33^

Some of the studies attempted to elucidate further details regarding the population of travelers, such as major reasons for travel, age, gender, time, and seasonality of travel. In the context of Ethiopia, for example, it is believed that most of the imported infections are due to routine human travel to areas of higher transmission intensity, with nonimmune people from high-altitude areas^15^ travelling to low-altitude areas during periods of high mosquito-biting (e.g., movement for harvesting crops).^14^ Similarly, the study in Kenya found the increased risk of malaria infection with travel to be concentrated in children aged < 5 years.^33^ Identifying characteristics of groups at high risk for travel and possibly importing infection should be a key area of focus for future studies to elucidate further knowledge gaps and enable the design of appropriate strategies for control.^2^

The relationship between malaria and travel is complex; nonetheless, imported malaria has potential for establishing transmission in the most receptive and vulnerable locations^3^ in addition to burdening the health system with imported malaria cases.^7^ It is likely that residents from non–malaria-endemic settings who travel to malaria-endemic settings have less access to information and interventions on malaria prevention, which further makes them more susceptible to infection during travel. On the other hand, although infected visitors from malaria-endemic areas to non-endemic areas may be better informed about prevention, it is likely that they have less access to malaria prevention interventions during travel. Therefore, given the optimal receptivity and vulnerability conditions, imported malaria has potential to reestablish and sustain transmission in low transmission settings. Moreover, evidence from Zanzibar suggests that imported malaria among returning residents was more important than infected visitors from Tanzania mainland.^34^ Therefore, given these complexities, tracking the at-risk populations and designing combinations of approaches to address malaria importation are needed to enable targeting residents travelling to malaria-endemic areas, residents returning from malaria-endemic areas, and visitors from malaria-endemic areas with appropriate interventions.^8^

This review has several potential limitations. First, travel history in all studies was based on self-reports, and there is, to our knowledge, no literature on establishing the validity and accuracy of this exposure in studied populations. In addition, self-reported travel history was likely to be influenced by recall bias, especially in studies that looked at travel history dating back to several months. Second, most of the studies were case–control studies, and they are inherently susceptible to selection bias. Third, our review only included studies undertaken in sub-Saharan Africa and where there was a priori expectation that travel would put a person at risk rather than one in which travel might reduce risk. Finally, although the analysis does not suggest any evidence of publication bias, it showed substantial heterogeneity in the magnitude of association across studies.

As more and more malaria-endemic countries aim to attain elimination of malaria, imported malaria has remained an important risk factor for reestablished transmission. The strength of the association implied by the random-effects meta-analysis pooled estimate and directional consistency of the study-specific findings support travel as an important risk factor for infection and, possibly, sustaining malaria transmission in malaria elimination settings. Therefore, national malaria control or elimination programs need to identify at-risk populations and travel patterns that drive malaria transmission and implement appropriate interventions to limit malaria importation and minimize its potential impact on autochthonous transmission.
